# The Role of Water-Soluble Vitamins and Vitamin D in Prevention and Treatment of Depression and Seasonal Affective Disorder in Adults

**DOI:** 10.3390/nu16121902

**Published:** 2024-06-17

**Authors:** Alireza Jahan-Mihan, Priscilla Stevens, Saily Medero-Alfonso, Georgina Brace, Laurel Kate Overby, Kristin Berg, Corinne Labyak

**Affiliations:** Department of Nutrition and Dietetics, University of North Florida, 1 UNF Dr., Jacksonville, FL 32224, USA; n01483548@unf.edu (P.S.); n01440556@unf.edu (S.M.-A.); n00835183@unf.edu (G.B.); n01242364@unf.edu (L.K.O.); k.berg@unf.edu (K.B.); c.labyak@unf.edu (C.L.)

**Keywords:** depression, seasonal affective disorder, treatment, vitamin B, C, and D, diet

## Abstract

Depression is a major global health concern expected to worsen by 2030. In 2019, 28 million individuals were affected by depressive disorders. Dietary and supplemental vitamins show overall favorable preventative and therapeutic effects on depression. B vitamins are crucial for neurological function and mood regulation. Deficiencies in these vitamins are linked to depression. Studies on individual B vitamins show promise in improving depressive symptoms, particularly thiamin, riboflavin, niacin, and folate. Vitamin C deficiency may heighten depressive symptoms, but its exact role is not fully understood. Seasonal Affective Disorder (SAD) is associated with insufficient sunlight exposure and vitamin D deficiency. Vitamin D supplementation for SAD shows inconsistent results due to methodological variations. Further investigation is needed to understand the mechanisms of vitamins in depression treatment. Moreover, more research on SAD and light therapy’s efficacy and underlying mechanisms involving photoreceptors, enzymes, and immune markers is needed. Although dietary and supplemental vitamins show overall favorable preventative and therapeutic effects on depression, dietitians treating psychiatric disorders face challenges due to diverse study designs, making direct comparisons difficult. Therefore, this article reviews the current literature to assess the role of dietary and supplemental vitamins in the prevention and treatment of depression. This review found that, although evidence supports the role of B vitamins and vitamins C and D in preventing and treating depression, further research is needed to clarify their mechanisms of action and determine the most effective intervention strategies.

## 1. Introduction

### 1.1. Depression

Depression is one of the top causes of disease burden worldwide [1,2,3,4]. Despite considerable advances in pharmacology and therapy techniques, the general population prevalence of depression has not declined [5,6]. In 2019, it was estimated that depressive disorders affected 280 million people [7]. The prevalence of depressive disorders varies across the world, but the 12-month prevalence estimated by the World Health Organization, World Mental Health (WHO, WMH) survey ranged from 2.2% to 10.4%, with a median of 5% across all countries [8]. Depression is a major public health concern identified as a national [9] and global priority [10].

While depression has been recognized as a major health burden, its definition is still debatable [4,11]. Depression is usually described by most psychiatrists as a disorder that is common [4,6,8,9,11], serious [8], debilitating [6,11], and potentially lethal [5,6,8,9,11], limiting psychosocial functioning [4,8,9], and generally diminishing the quality of life [4,6,8,11]. Depression has been recognized as a serious life modifier and has been identified as a contributing factor to reduced work productivity [12,13,14], increased substance abuse [15,16], family dysfunction [17,18], and reduced life expectancy [5,19]. Depressed individuals may experience one or more of the myriad symptoms that vary at different levels of intensity gradually but progressively across a lifetime. However, some individuals also may experience abrupt, sudden-onset and/or prolonged episodes of depression [20]. Additionally, most patients’ course of illness is episodic in nature, and a sense of homeostasis is experienced between acute depressive episodes [20]. Symptoms can be specific to a particular depressive disorder and can be categorized as neurovegetative, emotional, or cognitive. Neurovegetative symptoms, such as changes in appetite and sleep patterns, may follow various daily/seasonal patterns [4]. Those who suffer from depression tend to make associations between concepts of depression such as hopelessness and worthlessness resulting in intensified negative self-views [20].

Treatment of depression requires an interdisciplinary approach including psychological therapy, pharmacotherapy, or both [4,21,22]. Moreover, a properly balanced diet and appropriate supplementation are essential for optimal health. It is important to note that vitamins are more efficiently absorbed from dietary sources rather than from supplements. Although more than half of those affected by a Major Depressive Disorder (MDD) recover within six months, and two-thirds to three-quarters of those recover within a year, up to 27% of patients do not recover and develop a recurring depression [4,23]. Furthermore, those who have recovered can still experience residual symptoms such as a lack of energy and motivation [24].

### 1.2. Seasonal Affective Disorder (SAD)

Seasonal affective disorder (SAD) can be described as a form of recurrent depression in which the episodes occur annually, and the severity of the episode varies [25,26]. The prevalence of SAD is estimated to vary from 1.4 to 9.7% in Northern America and 1.3–3.0% in Europe [25], affecting more women than men [27], with a ratio varying between 3.5:1 and 9.0:1 [25]. When SAD was initially discovered, it was defined as a syndrome in which depression episodes developed during the fall/winter with remitting episodes during the spring or summer that lasted for at least two consecutive years [26]. Currently, the classification of SAD has been updated and is not considered a unique diagnostic entity; rather, it has been classified as a type of bipolar disorder or depression known as MDD with Seasonal Patterns in the Diagnostic Manual of Mental Disorders [25,28,29,30,31]. The first episode of seasonal affective disorder occurs around the age of 30, and symptoms are closer to non-seasonal depressive symptoms such as insomnia, poor appetite, weight loss, agitation, and anxiety [25].

The episodes of SAD are accompanied by common depressive symptoms and atypical autonomic nervous symptoms [26]. In comparison to SAD, other forms of recurrent depression occur throughout the year independent of the season. Furthermore, the intensity of the symptoms varies. Although SAD has been recognized as a psychiatric disorder and introduced into the Diagnostic and Statistical Manual (DSM), its identification as a valid and specific disorder remains controversial and the syndrome does not have a specific diagnostic category [29]. Currently, various questionnaires such as the Seasonal Beliefs Questionnaire (SBQ), Seasonal Health Questionnaire (SHQ), Seasonal Pattern Assessment Questionnaire (SPAQ), and the Seasonality Assessment Form have been used to determine whether a clinical diagnostic interview is needed [32]. The SPAQ is the most commonly used screening tool for SAD [31].

### 1.3. Diet and Micronutrients as Preventive and Therapeutic Approaches

Proper nutrient intake can alleviate some of the symptoms of depression and SAD. Supplementation of B, C, and D vitamins has been studied to alleviate depressive symptoms. Over the past two decades, there has been an increasing recognition of the role of diet and dietary bioactive components as a significant modifiable risk factor in the prevention and treatment of the wide spectrum of mood disorders including depression. Dietary nutrients, particularly certain vitamins, have shown promise in the prevention and improvement of depressive symptoms, regardless of individual self-efficacy and levels of physical activity [33,34,35,36,37].

Numerous observational studies have underscored the link between poor dietary habits and deteriorating mental health. Restricted diets including improperly planned diets that eliminate animal products may carry the risk of deficiencies in certain vitamins and minerals [38]. In contrast, certain nutrients have shown favorable effects by enhancing mood, which is closely aligned with the principles of the Mediterranean diet, featuring lean meats, fish, green leafy vegetables, legumes, and nuts. Notably, these food groups are abundant in vitamins, suggesting a possible mediation of the relationship between mood and dietary patterns through the intake of these essential micronutrients. Vitamins B, C, and D have been implicated in promoting brain health and mood stability, while deficiencies in vitamins B12, folate, or vitamin D are linked to elevated risk and occurrence of depression [33,34,35,36,37]. The current literature is not consistent in supporting the role of vitamins in the prevention and treatment of mood disorders and more specifically depression. Therefore, the purpose of this article is to review current studies and assess whether B vitamin, vitamin C, and/or vitamin D supplementation is instrumental as an adjacent treatment for depression and SAD (Table 1).

## 2. Methodology

The databases used to conduct this literature search were PubMed, UNF One Search through the UNF Library, and Google Scholar. The study types included randomized control trials (RCTs), prospective and retrospective studies, animal studies, cohort studies, and systematic reviews. A combination of keywords including B vitamins, vitamin C, vitamin D, depression, seasonal affective disorder, seasonal depression, randomized control trials, animal studies, clinical trials, winter blues, summer depression, light therapy, and vitamin supplementation were used to find relevant studies. The effect of supplementation of vitamins in adults 18 years of age and older with symptoms or at risk of depression was also investigated. The inclusion criteria encompassed all randomized controlled trials, animal studies, clinical trials, and systematic reviews investigating the role of B vitamins, vitamin C, and vitamin D in the prevention and treatment of depression, seasonal affective disorder, and seasonal depression in adults. Exclusion criteria included non-English publications, non-peer-reviewed articles, and studies with children as participants. Studies whose participants had metabolic abnormalities due to medical conditions including hyperparathyroidism, celiac disease, chronic pancreatitis, Crohn’s disease, and cystic fibrosis were excluded; studies in which the primary focus was another major psychiatric condition such as bipolar disorder, schizophrenia, and anxiety disorders, were also excluded.

## 3. Etiology of Depression and Seasonal Affective Disorder

The causes of depression remain unclear with possible etiologies being multi-factorial including low serotonin levels, stress, and inflammatory mechanisms. Several non-modifiable and modifiable factors, including genetics, environmental factors (such as life events, low social support, and financial problems), and lifestyle behaviors, including diet, may play critical roles in the incidence of depression and anxiety [39]. Notably, the role of food intake and dietary patterns in the development of mental diseases has been recognized [40,41,42]. Although difficulty regulating the neurotransmitter serotonin is believed to be responsible for balancing mood and abnormal melatonin metabolism was initially suggested as a potential underlying mechanism, more recent studies proposed inadequate exposure to sunlight as the main contributor to the etiology of SAD. Standard treatment includes antidepressant medications, light therapy, and vitamin D supplementation, along with counseling [30,31]. Both preventative and therapeutic effects of vitamin D supplementation on seasonal affective disorder (SAD) have been studied.

## 4. Vitamins and Depression

Comparing studies in this field has proven challenging due to the diverse study designs employed by researchers, including variations across intervention regimens (e.g., types, dosage, frequency, and duration), participants’ demographics (e.g., age range, gender, and health status), measured parameters, type of mood disorder, and overall study design.

### 4.1. B Vitamins

B vitamins are recognized as regulators of numerous immune functions, with deficiencies associated with various cancers. Moreover, they are referred to as “neurotropic” vitamins due to their neurospecific functions, playing significant roles in both the central and peripheral nervous systems [43]. The beneficial role of B vitamins in brain health and mood is well established [33,34,35] while their deficiencies (e.g., B12 or folate deficiency) have been linked to a higher risk and incidence of depression [36]. Consequently, there are calls for greater acknowledgment of nutritional deficiencies, or suboptimal nutrient status, as contributors to the underlying causes of depression and other psychiatric conditions [44,45]. However, research on the effect of dietary B vitamin intake on depression has been limited. Studies have explored both the collective therapeutic role of B vitamins and their individual effects. Furthermore, investigations have also examined how the status of B vitamins may affect the effectiveness of other therapeutic approaches. There is limited research on the therapeutic effects of dietary vitamins in depression, as most studies have primarily focused on vitamin supplementation. The primary focus of studies in the field was on the effects of B vitamins in clinically depressed, medicated participants [44,46]. Therefore, it is difficult to separate the direct effects of supplementation from those resulting from interactions between B vitamins and antidepressants. Although these results provide valuable insights into the effectiveness of B vitamins as an adjunctive therapy alongside medication, their results cannot be conclusive when it comes to the sole benefits of B vitamin supplementation alone. Additionally, due to altered structure and function of neural architecture in depression [47,48], it is difficult to understand the preventative effects of B vitamin supplementation in depression.

Moreover, the difference in the baseline of the vitamins and nutrients status in general is another factor that may influence the effectiveness of B vitamins in the treatment of depression and other mood disorders [49]. There is increasing evidence that suboptimal nutrient status, below clinical deficiency criteria, may be a risk factor for poor functional status [45,50]. While it is crucial to determine whether individual B vitamins are effective, it is also critical to consider these findings in the context of baseline nutrient status, dietary habits, and co-administration of other nutrients as part of the intervention.

Many studies implemented a group of B vitamins as a therapeutic approach in their studies, and this can be due to the interdependence of B-group vitamins within the methylation cycle [12,34]. The polymorphism in methyltetrahydrofolate reductase (MTHFR) is one of the factors determining the effectiveness of B vitamin therapy in depression. Therefore, a combination of B-group vitamins should be more effective than supplementation with B6, B12, or folate alone, as they are all involved in methyl metabolism.

#### 4.1.1. Vitamin B1 (Thiamin)

Vitamin B1, also known as thiamin, plays a critical role in the function of the neurological system. Thiamin deficiency has been associated with mood disorders, anxiety, and depressive symptoms [51]. Historically, thiamin was initially named aneurin due to its connection with “beriberi” resulting from dietary deficiency of thiamin. In developing countries, thiamin deficiency continues to be a prevalent issue, primarily attributed to the widespread consumption of white rice since it lacks bran which is the source of thiamin [52]. Adequate levels of thiamin in the diet during development is crucial, as a deficiency can lead to significant cognitive impairments by the age of five, due to impaired brain development [53]. In a cross-sectional study on 9848 adults, 4.38% were diagnosed with depression. Vitamin B1 and B3 intake mixture were negatively associated with depression [54]. Similarly, in a study on 34,700 participants, thiamine intake was strongly associated with lower risks of hypertension, type 2 diabetes, depression, and dyslipidemia [55].

While there is a substantial body of evidence linking dietary thiamin status to neurological functions, there is a limited number of studies that have specifically investigated its therapeutic effects in mood disorders and depression. In one study, a correlation between poor thiamin nutritional status and an increased likelihood of depressive symptoms was shown [56]. In this cross-sectional study involving over 1500 elderly Chinese individuals aged 50 to 70 years, a correlation between lower levels of erythrocyte thiamine (likely indicative of thiamine pyrophosphate (TPP) bound to transketolase (TKT)) and increased severity of depressive symptoms were observed. Moreover, previous research identified a direct correlation between thiamine deficiency and symptoms of major depressive disorder (MDD) in a sample of 74 individuals with a history of malnutrition [57]. These results were further supported by another study involving 118 geriatric patients [58], although no data regarding dietary thiamine intake were provided. Supplementation of thiamin has shown promising results in numerous studies. The therapeutic effects of thiamin supplementation as a palliative measure for postpartum depression (PPD) look promising and may play a crucial role in the subsequent cognitive development of the infant. In elderly women with marginal thiamin deficiency, a six-week supplementation led to enhanced general well-being, increased activity and energy levels, improved sleep patterns, and alleviation of depression symptoms [59]. Moreover, Nguyen et al. reported thiamin and niacin intake had negative trends and was observed as the most important factor negatively associated with depression [55]. In another study, a co-supplementation of thiamin with riboflavin and pyridoxine in geriatric patients resulted in improved depression and cognitive function scores compared to a placebo group [60]. Moreover, the role of thiamin supplementation as an adjuvant therapy for patients with MDD has also been studied. In a study including patients, administration of thiamin alongside conventional treatment led to noticeable improvements in depressive symptoms within six weeks [61]. In mouse-induced PPD, improvement in depressive symptoms and anxiety-related behaviors, assessed through tests such as the forced swimming test and elevated plus-maze, were observed in the mothers following thiamin, zinc, and magnesium administration on postpartum day 3. Additionally, there was an increase noted in the total antioxidant capacity [62]. These findings collectively suggest that thiamin deficiency contributes to depression and that supplementation can effectively mitigate associated symptoms.

#### 4.1.2. Vitamin B2 (Riboflavin)

The association between riboflavin and depression has been studied [40,63,64,65]. Previous studies have shown an inverse relationship between riboflavin and psychological disorders [66,67]. Dairy products are the primary source of riboflavin. In a more recent study, higher intake of riboflavin showed a protective effect against depression [65]. Similarly, a cross-sectional study including adult populations showed reduced odds of depression with the highest riboflavin intake [67]. In another cross-sectional study including psychiatric inpatients, a significant inverse relationship between riboflavin intake and endogenous depression was observed [66]. In a cross-sectional study on 98 female clinical nurses (45 depressed), marginal riboflavin deficiency was more prevalent in depressed subjects [68]. In this study, depression status was assessed using the Beck Depression Inventory.

Additionally, a study including 6517 adolescents showed that B2 intake was inversely associated with depressive symptoms in females but not males [40]. In an observational study, the mental component score (MCS-12) was positively correlated with vitamin A, vitamin C, riboflavin, and folate [43]. Moreover, a meta-analysis of six epidemiological studies showed an inverse association between riboflavin intake and depression [69]. In a cross-sectional study on 314 HIV–infected adults, approximately 26% of the participants experienced depression [70]. Moreover, more than 67% of the individuals consumed B vitamins below the estimated average requirements (EAR).

In this study, low riboflavin consumption was associated with a higher risk of depression in females but not males. While the exact mechanism remains unclear, it has been suggested that female gonadal hormones may alter serotoninergic activity in the brain, affecting the regulation of monoamine levels such as serotonin and leading to higher levels of depressive symptoms [71].

#### 4.1.3. Vitamin B3 (Niacin)

Niacin is a nutritional precursor to nicotinamide adenine dinucleotide phosphate (NADP) and nicotinamide adenine dinucleotide (NAD), which are essential cofactors for mitochondrial energy metabolism [72]. Niacin both plays a major role in neuroprotection and is vital for the normal functioning of the central nervous system (CNS) and neuronal development [73]. The relationship between niacin intake and depression has been studied extensively. Altered synthesis of niacin metabolites can lead to various neuropsychiatric disorders [74]. Niacin deficiency may lead to reducing oxidative phosphorylation and impairs mitochondrial respiration [75]. The trinity of brain energy deficit, mitochondrial dysfunction, and oxidative stress might exert a role in the development of depression [76,77]. Indeed, niacin supplementation improves symptoms of anxiety, irritability, and depression [78,79]. Moreover, niacin may cure NAD deficiency [75]. Antidepressants may lead to a decrease in niacin and NAD in patients with poor intake [80].

Nephrotic syndrome or chronic diarrhea can lead to niacin deficiency despite regular intake [81]. However, the response to niacin therapy can be varied due to the variation in genetic background. Niacin has also been used as a diagnostic tool. Niacin affects the skin and is activated via the arachidonic acid–cyclooxygenase pathway. This activation can lead to skin flushing symptoms, known as the niacin skin flushing reaction. This reaction may indirectly reflect the level of oxidative stress in the body and could hold potential diagnostic value in psychiatric disorders [82]. The niacin response biomarker may serve as a schizophrenia endophenotype [83].

Additionally, the “niacin skin blunting response” has high diagnostic value in adolescent patients with schizophrenia or bipolar disorder [84]. Moreover, niacin skin sensitivity is elevated in adolescents at risk for psychosis [85]. In one study, thirty-eight cases of acute episodes of depression in unmedicated adolescents and 47 age- and sex-matched healthy controls were included, all of whom were stimulated with six concentration gradients of niacin solution on the forearm skin, and the skin flushing area was applied as an assessment index. The total area of redness of the skin in response to niacin was significantly lower in the adolescent depression group than in the healthy adolescent group [83]. However, in another study, when patients diagnosed with schizophrenia (*n* = 16), and depression (*n* = 16) were compared with healthy controls, no significant difference in skin flushing between depressed patients and control was observed, but the difference between schizophrenic patients and the control group was striking [86].

#### 4.1.4. Vitamin B5 (Pantothenic Acid)

Vitamin B5, also known as pantothenic acid, is frequently regarded as an ‘antistress factor’ and plays a crucial role in the normal development of the central nervous system. It contributes to the synthesis of acetylcholine, a neurotransmitter associated with depression. Deficiency in pantothenic acid can result in fatigue and depressive symptoms. Research indicates a correlation between low pantothenic acid levels alongside other B vitamins’ deficiency and depressive symptoms in school children aged 6–9 years [87]. Furthermore, there is evidence suggesting that higher nutrient intake, including pantothenic acid, is linked to improved mental health outcomes such as mood, depression, and mania scores [88]. Consequently, further studies on nutrient intake, particularly pantothenic acid, are warranted to explore its potential as a treatment option for individuals with mood disorders, anxiety, and depression.

#### 4.1.5. Vitamins B6, B9, and B12 (One-Carbon Metabolism)

Three B vitamins, B6, B12, and folate, are known as cofactors in one-carbon metabolism. The essential role of these B vitamins in one-carbon metabolism may link B vitamin status with mood [89]. It may explain why a complete spectrum of B vitamins contributes to the interconnected cellular processes responsible for homocysteine homeostasis and DNA methylation. Therefore, deficiencies in one or more of these vitamins may impair methyl metabolism that may lead to hyperhomocysteinemia [90]. Elevated homocysteine is a risk factor for not only cardiovascular diseases but also for poor mood. There is a line of evidence supporting the link between hyper-homocysteinemia and poor mood [91]. In one study, up to 30% of depressed patients have elevated homocysteine levels [91]. Therefore, while deficiency of these vitamins may increase the risk of mood disorders, supplementation with B group vitamins including B6, B12, and folate aimed at lowering homocysteine levels would be expected to support improvements in mood outcomes.

#### 4.1.6. Vitamin B6 (Pyridoxine)

Vitamin B6, comprising pyridoxine, pyridoxal, and pyridoxamine, plays a crucial role in mood regulation and is strongly implicated in both the onset and management of depression [92]. The role of vitamin B6 (pyridoxal phosphate, PLP), another B vitamin involved in one-carbon metabolism, has also been investigated. PLP plays a crucial role in the synthesis of neurotransmitters including noradrenaline, serotonin, and gamma-aminobutyric acid (GABA) [93], thus affecting mood and anxiety levels. Deficiency in this vitamin can lead to elevated homocysteine levels, which have been associated with seizures, migraines, and depression. Pyridoxine supplementation has been shown to lower blood plasma homocysteine levels in patients with schizophrenia and improve accompanying mood disorders, including minor depression [94,95]. An association between low serum pyridoxal levels and major depression in 18 individuals exhibiting depressive symptoms is reported [96]. PLP exhibits positive effects on overall health, mood, and cognition [33]. Moreover, in females, PLP supplementation (100 mg/day) has shown favorable effects in improving premenstrual symptoms and depression [97].

Furthermore, higher intakes of vitamin B6 and B12 may reduce depression in older adults [98]. A combination of magnesium (300 mg/day) and vit B6 (30 mg/day) supplementation for eight weeks resulted in significantly lower Depression Anxiety Stress Scales (DASS-42) stress subscale scores in adults diagnosed with depression [99].

#### 4.1.7. Vitamin B9 (Folate)

The link between depression and folate deficiency is well documented [100,101,102]. Folate deficiency increases the vulnerability to depression [103,104]. Moreover, folate deficiency exacerbates depressive symptoms [102], and prolongs depressive episodes [105]. In a meta-analysis, researchers found that individuals with depression had lower folate levels than those without depression [106]. Consistently, in another meta-analysis, a significant association between low folate and B12 levels and depression was observed which was also gender-dependent: A positive association between low B12 but not folate levels and depression only among women was observed [107]. Finally, the risk of depressive symptom relapse may also increase in cases diagnosed with folate deficiency [108]. Several underlying mechanisms by which folate status is linked to depression and other mood disorders have been suggested: First, the role of folate in the synthesis of S-adenosylmethionine (SAM) is one of the major mechanisms by which folate is connected to the depression [100,101,102,103,104]. S-adenosylmethionine has a key role in the synthesis of dopamine, norepinephrine, and serotonin, the neurotransmitters with a strong link to depression. SAM is also one of the regulators of tetrahydrobiopterin synthesis, a cofactor in neurotransmitter synthesis [100,105,109,110]. Folate deficiency may lead to decreased levels of dopamine, norepinephrine, and serotonin, thereby creating a neurochemical predisposition to depression. Second, elevated homocysteine levels due to folate deficiency is another potential mechanism by which folate status may link to depression and other mood disorders, since a strong positive correlation between elevated homocysteine levels and the severity of depressive symptoms has been reported [91,103]. A correlation between low serum and/or red blood cell (RBC) folate levels and elevated plasma homocysteine concentrations is well established. A strong correlation between low folate levels in individuals with depressive disorders has been consistently identified. Shorvon et al. [111] reported folate deficiency in 56% of patients with affective disorders. It was consistent with AbouSaleh and Coppen’s [112,113] observations indicating that patients diagnosed with major depressive disorder had significantly lower serum and RBC folate levels compared to healthy controls. Furthermore, reduced serum folate concentrations were associated with increased depression severity. Carney et al. [114] discovered that RBC folate levels were notably lower in depressive patients compared to those with other psychiatric conditions. Additionally, associations between disease severity, duration, and folate status have been observed. A recent study by Morris et al. [115] identified low folate status in depressed individuals aged 15–39 within a general US population sample. Finally, genetic factors affecting folate metabolism may impact the risk of depression. Methyltetrahydrofolate reductase (MTHFR) facilitates the conversion of methyltetrahydrofolate, thus exerting a significant indirect impact on homocysteine remethylation. An association between genetic factors that impair the metabolism of homocysteine and depression is reported. A link between the C677T TT genotype of the MTHFR enzyme and depression has been reported as the carriers of the TT genotype are 1.37 times more likely to be diagnosed with depression compared to those with the CC genotype [116]. In another study on patients with major depression, the prevalence of the TT genotype was more than double in patients compared to controls [117]. In another study, anxiety and depression were measured in 5948 subjects within two age-range groups (46–49 and 70–74 years old) in Norway. A strong positive correlation between plasma homocysteine levels and increased risk of depression was observed.

The TT genotype of the MTHFR gene was also associated with an increased risk (69%). However, low plasma folate was correlated to depression only in 46–49-year-old women [118]. Folate status may also influence the therapeutic outcomes. The therapeutic efficacy of both sertraline, a selective serotonin reuptake inhibitor (SSRI), and nortriptyline, a tricyclic antidepressant, is dependent on the baseline RBC folate levels. In geriatric depression, a higher folate status predicted a more favorable outcome, with the association being particularly pronounced for sertraline [119]. Interestingly, antidepressant therapy has been found to coincide with an elevation in RBC folate levels, contingent upon both folate and vitamin B12 status. Moreover, this increase was more significant in responders compared to non-responders [120]. The therapeutic properties of SAM alone or along with antidepressants in the treatment of depression are well documented [121,122]. Both folate and B12 are involved in SAM synthesis. The therapeutic effects of folate supplementation on depression received considerable study. Folic acid exhibited antidepressant-like effects in open-field and sucrose preference tests. Folic acid treatment effectively increased the levels of monoamine neurotransmitters, BDNF and β-endorphin, and interleukin-6, and homocysteine levels were also significantly suppressed by folic acid administration [123]. The antidepressant effect of folate is well documented. In chronic unpredictable mild stress-induced Sprague–Dawley rats, folate supplementation exhibited antidepressant-like effects in open-field and sucrose preference tests. It increased the levels of monoamine neurotransmitters, and homocysteine levels were suppressed [123]. In another study, supplementation of 15 mg of folic acid on a daily basis improved symptom relief and social adaptation in severely depressed patients with low RBC folate levels (<200 ng/mL) compared with control group [124]. In another study, the effect of folate was compared with trazodone; both 50 mg of folic acid and 100 mg daily of trazodone showed comparable effects. Notably, in this study, RBC folate levels were within normal range [125]. Consistently, in another clinical study, supplementation with methyl folate (15 mg/day for 6 months) enhanced the therapeutic effects of standard psychotropic treatment in 41 patients with major acute depression or schizophrenia. The RBC folate levels were low at baseline [126]. The synergic effect of folate with other medications has also been investigated. In a study on depressive patients, supplementation of 0.5 mg/day of folic acid along with 20 mg/day of fluoxetine improved the therapeutic effect of fluoxetine by 61.1% compared with fluoxetine alone in the placebo group. In this study, a decrease of over 50% in Hamilton rating scale scores for 93.3% of patients was observed. Moreover, folate reduced the homocysteine levels significantly (by 20.6%) only in women [109]. In a meta-analysis of three clinical trials, Taylor et al. found that folate may boost the effect of antidepressants by reducing Hamilton Depression Rating Scale (HDRS) scores on average by a further 2.65 points [102]. The effect of a combination of folic acid, B6, and B12 on symptoms of depression has been also studied. In a randomized, placebo-controlled trial, supplementation of daily folic acid (2 mg), vitamin B6 (25 mg), and vitamin B12 (0.5 mg) for 1 to 10.5 years in survivors of stroke resulted in a lower hazard of major depression compared with the placebo group (18.4% vs. 23.3%) [127]. In summary, substantial evidence now indicates a reduction in plasma folic acid levels, and it could be due to hyperhomocysteinemia associated with folate deficiency among individuals with depression. However, the favorable effect of folate supplementation on depression and other mood disorders is not clear and needs further study.

#### 4.1.8. Vitamin B12 (Cobalamin)

The role of vitamin B12 in the etiology and treatment of depression has also been investigated. Vitamin B12 is a water-soluble vitamin primarily synthesized by bacteria in the human body. Insufficient intake of this vitamin can result in symptoms such as fatigue, weakness, constipation, balance problems, mental fog, peripheral tingling, depression, and cognitive impairments [128,129]. Moreover, lack of Castle intrinsic factor may lead to vitamin B12 deficiency despite sufficient intake. It can be due to atrophic gastritis, or AIG—autoimmune gastritis [130]. Vitamin B12, in conjunction with folate and vitamin B6, plays a crucial role in one-carbon metabolism. Consequently, the numerous functions associated with folate regarding depression and other mood disorders can also be extended to encompass vitamin B12. Vitamin B12 deficiency has a major role in the etiology of depressive symptoms [131].

Deficiency of vitamin B12 in patients diagnosed with depression has been well documented [132,133,134,135]. In a study in the Indian population, neurological problems were more prevalent in vegetarians, with poor vitamin B12 status [135]. Pregnant women with vitamin B 12 deficiency were more prone to depression which was accompanied with hyperhomocysteinemia [136]. Hyperhomocysteinemia due to vitamin B12 deficiency is one of the key mechanisms connecting vitamin B12 to depression and other mood disorders. The effect of supplementation of B12 in the treatment of depression has also been studied [137]. Higher consumption of vitamin B12 and B6 was associated with a lower risk of depressive symptoms [98]. In a randomized controlled trial, daily supplementation with oral vitamin B12 (100 mcg) and folic acid (400 mcg) resulted in an increase in cognitive function [138]. Moreover, antidepressant therapy corresponded to an elevation in red blood cell (RBC) folate levels, with responsiveness. Folate levels in RBCs are dependent on both folate and vitamin B12 statuses. This increase was notably more pronounced in responders compared to non-responders [139].

#### 4.1.9. Vitamin B7 (Biotin)

Vitamin B7 is essential for cell growth and the metabolism of fats and amino acids. In cases of severe depression and delirium, treatment with biotin has shown improvements in symptoms [140]. Inherited biotinidase deficiency in newborns can lead to neurological abnormalities [141]. In a clinical trial, individuals diagnosed with depression either received a multi-strain probiotic plus biotin treatment or biotin plus placebo for 28 days [142]. Psychiatric symptoms were improved in both groups. The number of studies on the role of biotin in depression is extremely limited. Further research is needed to fully understand its role in depression. The interactive effects of biotin and microbiota on symptoms of depression have been studied. In a clinical study, 82 individuals diagnosed with depression were randomly divided into two groups. One group received a multistrain probiotic along with biotin treatment, while the other group received biotin alongside a placebo for a duration of 28 days. Both groups showed significant improvement in psychiatric symptoms. However, the probiotics group exhibited a higher abundance of Ruminococcus gauvreauii and Coprococcus, as well as increased β-diversity. KEGG analysis revealed heightened activity in inflammation-regulatory and metabolic pathways within the intervention group. The authors suggested that the beneficial effects observed with probiotic treatment combined with biotin may indicate the effectiveness of probiotic therapy in individuals with depression [142].

### 4.2. Vitamin C (Ascorbic Acid)

Vitamin C plays a crucial role as an antioxidant in the brain and serves as a co-factor in the synthesis of neurotransmitters including adrenaline and other neurotransmitters [143,144]. The central nervous system (CNS) is vulnerable to oxidative damage due to an abundant amount of polyunsaturated fatty acids and transition metals when the levels of endogenous antioxidants in CNS is relatively low [145,146]. Oxidative damage in the CNS has strongly been associated with anxiety disorders [147], psychosocial stress [148], and depression [149,150,151,152]. The need for vitamin C in excessive stress is higher to sustain normal brain function which is due to the disproportionate utilization of nutrients during stress responses, essential for synthesizing stress hormones, alongside higher demands for cellular energy production [153,154,155]. Lower plasma concentrations of antioxidants, including ascorbate [156], α-tocopherol [157], and antioxidant enzymes [158,159] are reported in depressive individuals which may explain the increased levels of lipid peroxidation in these individuals [160,161]. The lower plasma concentrations of antioxidants are accompanied by elevated levels of lipid peroxidation end products in patients diagnosed with anxiety disorders [160,161]. Moreover, the synthesis of neurotransmitters like serotonin, which regulate mood, appetite, and sleep, in chronic stress may compromise as priority shifts towards producing survival hormones rather than those that promote stability, calmness, and restful sleep [153,154]. The impact of vitamin C supplementation (1000 mg/day) on stress and anxiety by assessing the effects of high-dose sustained-release vitamin C (1000 mg) supplements in a group of 60 young adults over 14 days was assessed. The authors suggested that supplementation of a high dose of vitamin C, but not dietary vitamin C, yields anxiety- and stress-lowering effects. In another clinical study, researchers found that chronic social isolation stress induces weight gain and depressive-like behavior, but this effect is diminished by vitamin C [162]. In mice, depressive-like behavior and hippocampal synaptic dysfunction induced by corticosterone promptly reversed with a single administration of vitamin C [163].

It has been suggested that there is a potential link between clinical depression and the onset of scurvy [164]. Depletion of vitamin C has also been linked to heightened depressive symptoms [165]. A potential relationship between vitamin C deficiency and depressive symptoms is proposed in a case study. A 52-year-old colon carcinoma patient developed scurvy, accompanied by decreased appetite, fatigue, weakness, anhedonic behavior, and depression. Subsequently, supplementation with vitamin C (1 g/day for 7 days, followed by 1.5 g/day for 7 days, orally) resolved both the scurvy and depressive symptoms in this patient [166], consistent with findings from previous literature [167,168,169]. Nevertheless, gulo knockout mice deprived of vitamin C did not show any significant change in immobility time in the tail suspension test, in contrast to gulo knockout mice that were supplemented with vitamin C [170,171]. Although the underlying mechanisms by which vitamin C deficiency is connected to depression are poorly understood, it has been suggested that chronic vitamin C deficiency can result in reduced activity of the enzyme dopamine β-hydroxylase, thereby impairing the hydroxylation of dopamine (DA) to norepinephrine (NE) and consequently lowering NE levels [171].

### 4.3. Vitamin D (Calciferol)

Vitamin D (calciferol) is a fat-soluble vitamin that is obtained from (1) ultraviolet (UV) irradiation to the skin that stimulates a conversion of cholesterol (7-dehydrocholestrol), (2) plant-based foods in the form of D2 (ergocalciferol), and (3) animal-based foods in the form of D3 (cholecalciferol) [172,173,174,175]. Vitamin D, upon ultraviolet exposure or dietary ingestion, metabolizes to its active forms of calcidiol [25-hydroxyvitamin D (25(OH) D)] in the liver and calcitriol [1,25- dihydroxyvitamin D (1,25(OH)2 D)] in the kidneys and other tissues for communication between cells and hormone signaling [172,173,174,176].

As a multifunctional hormone, calcitriol is involved in calcium homeostasis by promoting calcium absorption in the gastro-intestinal (GI) tract to prevent hypocalcemia tetany [172,173]. Other functions of vitamin D include reducing inflammation (anti-inflammatory), cell growth, neuromuscular and immune function, and glucose metabolism [172]. The photochemical conversion of 7-dehydrocholesterol to vitamin D3 via UV irradiation can be influenced by various factors including altitude, season, time and length of the day, clouds, and smog. An average of twenty minutes per day of sun exposure provides 50–90% of the daily vitamin D recommendation, and the remainder can be obtained from dietary sources of vitamin D [177]. Fatty fish, dairy, liver, eggs, soy, and fortified foods are good dietary sources of vitamin D; however, the bioavailability of vitamin D from animal sources is more effective at raising serum 25(OH)D concentrations than plant-based food sources [173,178].

A factor that may affect vitamin D efficacy includes the use of topical sunscreen and UV–protective clothes hindering vitamin D conversion. Age may also play a role as older adults decline in absorption rate of calciferol within the GI tract and through skin [177,179]. Darker skin pigmentation has higher melanin content which reduces vitamin D synthesis through sunlight exposure [177,179]. Body weight, body composition, and health conditions may also influence vitamin D status [179]. Gastrointestinal disorders (e.g., Crohn’s, celiac disease, malabsorption syndrome such as lactose intolerance, and short bowel syndrome) have shown a negative impact on vit D absorption [177,179]. Thompson et al. indicated that malabsorption was evident with orally administered vitamin D3 in patients diagnosed with celiac disease, biliary obstruction, or pancreatic dysfunction [180]. Gastric bypass surgery, liver and kidney disease, hyperparathyroidism, sarcoidosis, tuberculosis, histoplasmosis, and lymphoma cancer are among other health conditions that diminish vitamin D absorption [177]. Vitamin D drug interactions may also affect vitamin D status. Medications that activate the pregnane X receptor (PXR) can interfere with vitamin D-responsive elements (VDRE) at DNA transcription and affect the expression of genes that are ordinarily regulated by vitamin D [181]. Glucocorticoids, bisphosphonates, anti-estrogens, cytostatic agents, HMG-CoA-reductase inhibitors, and antituberculotic drugs may also disrupt vitamin D metabolism and vitamin D functions [181].

#### 4.3.1. Vitamin D and Depression

The association between vitamin D supplementation and depression episodes has been extensively studied; however, the results are inconsistent. In a randomized control trial, participants (*n* = 18,353) who were at risk of incident depression or recurrent depression were given 2000 IU/d of vitamin D3 as a supplement for a period of 5.7 years. The treatment with vitamin D3 did not result in a statistically significant difference compared with the placebo in the incidence and recurrence of depression or clinically relevant depressive symptoms or change in mood scores [182]. Similar results were observed in a double-blind randomized controlled clinical trial including 152 participants supplemented monthly with 50,000 IU vitaminD3 for six months [183]. Although lower levels of plasma vitamin D in participants with higher depressive symptoms were reported in both studies, no therapeutic effect of high vitamin D intake and supplementation on depressive symptoms was observed, which was consistent with the results reported by another study where participants presented both low levels of vitamin D and major depressive disorders [184]. In comparison, significant improvements in well-being were reported by all participants in a randomized control trial [185]. In this study, 164 participants were randomly assigned to two groups and received either 15 or 100 mcg/day of vitamin D for six months. The group supplemented with 100 mcg/day of vitamin D reported higher levels of “well-being” than the group supplemented with 15 mcg/day. Two questionaries were used to assess the participants’ “well-being”, the first of which included a series of questions about mood, sleeping patterns, and energy levels. The second set of questions focused on other aspects of well-being, such as appetite, social factors, and general health. These questions were used to assess participants’ overall “well-being” [185].

In an observational study, vitamin D-deficient individuals had 3.5 times higher odds of developing clinically significant depression in comparison to those with sufficient vitamin D after adjusting for confounding variables [186]. However, the beneficial effect of vitamin D on depression was not corresponding to the recommended nutritional level defined as RDA in previous studies while higher concentrations above RDA might have a more significant therapeutic effect compared with the recommended level. Consistently, a significant correlation between lower vitamin D3 levels and clinically depressive symptoms was observed in three other studies [186,187,188]. Participants in these studies had similar age groups (18 years and above) and observed a positive relationship between vitamin D deficiency and clinically significant depression. In spite of different outcomes, the consistent observation throughout these studies is that while monthly supplementation or a single high dose results in a higher mean of serum 25-hydroxyvitamin D, a daily lower dose maintains the levels of serum 25-hydroxyvitamin D for a longer period [182,183,187]. Additionally, in the studies where participants received a single high dose of vitamin D, significant improvement in depression symptoms was observed, which further supports the idea that higher doses of vitamin D above RDA might have a positive therapeutic effect on depressive symptoms in those with insufficient levels of or deficiencies in vitamin D [189].

The inconsistency in the results of the studies can be explained by the various supplementation protocols (oral/intravenous; IV) applied and the difference in the forms of vitamin D dosages and the frequency of administration. In a randomized control trial, patients with low 25-hydroxyvitamin D levels were given 40,000 IU vitamin D orally per week for 6 months, in comparison to another randomized control trial where the participants received 1 single dosage of 150,000 or 300,000 IU of vitamin D via IV injection [185,189]. Both studies assessed the effect of vitamin D supplementation on SAD symptoms, but their supplementation protocols (oral/IV) and dosage were different. As a result, higher levels of improvement in depression symptoms were observed in the randomized control trial, where participants received 300,000 IU and 150,000 IU of vitamin D via IV [189]. In the study where the participants received 40,000 IU vitamin D orally per week for 6 months, no significant improvement in depressive symptoms scores was reported [185].

Moreover, in some studies, no placebo control groups are included, and therefore, it is difficult to interpret the results. The difference in the length of the studies, from 8 weeks to 1 year, and variation in the general characteristics of participants (e.g., gender and age range) are other factors that may also explain the inconsistencies in results. Limitations reported by these studies may also contribute to the variations in the results observed between the studies. For example, self-reported questionnaires of health behaviors and depressive symptoms, including participants with no vitamin D deficiency or who were not at risk of depression or had no symptoms of depression, limited information about participant adherence, no clinical interview to establish a diagnosis of depression, no screening for health factors that may affect vitamin D levels, and sample errors (samples not representing the overall population) are among them [177,182,183,184,185,186,188,189]. Additional confounders include socio-demographic and socio-economic status, biological and lifestyle factors associated with depressive symptoms, and vitamin D status.

Dietary interventions have long been known as an effective tool in the prevention and treatment of depression [184,190,191,192]. Prior research on the link between diet and depression has mostly focused on minerals, foods, and food groupings rather than dietary patterns; however, in studies, nutritional epidemiologists have proposed using a dietary pattern approach to investigate diet–disease relationships due to the intricate interactions between nutrients and foods [174,182,193]. Some studies have focused on the link between dominant eating habits and depression risk, driven by food questionnaires rather than biochemical testing [194,195,196]. After adjusting for confounding variables, a substantial link was identified between depression and serum vitamin D, as well as unhealthy eating patterns defined as under- or over-eating while having enough healthy foods to consume each day [175,194]. Vitamin D also functions as the mediator link between a poor diet pattern and depression [197]. Potential biomarkers, including serum concentrations of 25(OH) D, total antioxidant capacity (TAC), zinc (Zn), and magnesium (Mg) in serum, are reported to be significantly related to depression [175,194,197,198]. The correlation between depression and unhealthy eating is prevalent [194,195,196,197]. In a case-control observational study which included 110 depressed patients and 220 healthy individuals, serum vitamin D, zinc, magnesium, and total antioxidant capacity were considered potential mediator variables when evaluating the relationship between dietary patterns and depression [194]. The results showed that healthy and unhealthy dietary patterns were associated with lower and higher odds of depression, respectively, and there was a reverse relationship between serum vitamin D concentrations and depression after adjusting for potential confounders [194]. The study also showed that unhealthy dietary patterns were related to depression via altering the serum vitamin D concentration [186].

Another study has also shown that depression is inversely correlated with high protein and low fat intake and positively correlated with vitamin D-binding protein (VDBP) concentration when interacting with two polymorphisms (rs7041 and rs4588) in the GC gene, which encodes VDBP [199,200,201,202,203]. The role of the VDBP is to bind and transport vitamin D and its metabolites; this protein is positively correlated with depression due to the connecting effect of calcitriol on depression. Calcitriol (1,25[OH]2D3) helps to maintain both the number of neurons and the structure of neurons through detoxification mechanisms such as inhibiting inducible nitric oxide synthase synthesis, increasing glutathione levels, and regulating neurotrophin synthesis, all of which are factors influencing the risk of depression. The presence of vitamin D receptors, vitamin D-binding protein (VDBP), and/or the 1-alpha-hydroxylase enzyme, which converts 25(OH)D3 to 1,25(OH)2D3 in the brain, may explain the effect of calcitriol on depression [195]. In this study, 265 individuals (126 males, and 139 females) ages 18–55 were recruited from Tehran, Iran. Using the Depression Anxiety Stress Scales 21 (DASS-21) questionnaire, depression symptoms were classified as normal, moderate depression, or severe depression [199]. Participants were allocated into three groups: high-protein/low-fat diet, moderate-protein/moderate-fat diet, and low-protein/high-fat diet. An analysis of two polymorphisms in the GC gene was performed. A significant association between depression and diet was observed. The findings showed that depression was related to both the rs7041 and the rs4588 polymorphism [199,200,201,202]. Moreover, an interaction between a high-protein/low-fat diet and the rs7041 polymorphism, which has a greater prevalence in moderate and severe depression, was observed [199,200,201,202].

#### 4.3.2. Vitamin D deficiency and SAD

Dietary vitamin D intake is shown to not only have an inverse correlation with depression severity but also may have similar correlation with SAD [31,204,205]. Low serum 25-hydroxyvitamin D levels (25(OH)D) have been associated with a higher likelihood of SAD [198,199,201,205,206]. There is no strong evidence supporting the therapeutic role of dietary vitamin D intake in patients diagnosed with SAD, yet vitamin D supplementation may alleviate symptoms associated with SAD [188,203,207,208]. However, no studies have found a direct link between dietary vitamin D intake and SAD [203]. Future research is needed to examine the therapeutic role of dietary vit D intake in patients diagnosed with SAD and whether this effect is dose dependent. The sun’s exposure is one of the major determinants of vitamin D status and can be affected by weather, season, and the environment [177]. This seasonal factor emphasizes the link between vitamin D bioavailability and the prevalence of SAD. Unlike depression, individuals diagnosed with SAD tend to experience vegetative symptoms seasonally, particularly during the winter [24,27,29]. This trend has been highly associated with a reduction in light exposure during the winter [209,210,211,212,213]. The concept that SAD is a result of a lack of light exposure opens a window for the potential role of vitamin D deficiency as part of the etiology [26,214]. This effect can be explained by the role of vitamin D in serotonin synthesis. As previously described, cutaneous provitamin D3 7-dehydrocholesterol can be converted to previtamin D3 under UVB exposure [25,215]. Vitamin D in turn activates tryptophan hydroxylase 2 (TPH2) which is a neuronal-specific enzyme responsible for serotonin synthesis in the brain [205,216]. Low levels of serotonin are linked to depression, which could explain the cause of seasonal depression during the autumn and winter seasons [217]. However, unlike the clear link between vitamin D status and depression, the causal relationship between vitamin D status and seasonal depression is still unclear and open to further discussion.

As previously explained, seasonal affective disorder (SAD) can be mainly attributed to a lack of light exposure, but regulation of the neurotransmitter serotonin and the overproduction of melatonin also play key roles in impacting symptoms of SAD. Consequently, vitamin D is involved in the pathways of production of melatonin and is believed to play a key role in serotonin activity, which further links serotonin, melatonin, and light exposure, but mainly deficiency of vitamin D and SAD [218]. Therefore, it can be speculated that insufficient levels or a deficiency in vitamin D synthesis owing to a decrease in sunlight exposure can increase the risk of developing SAD [31].

Gender roles correlate with the prevalence of a potential impact on the etiology of SAD, according to The National Health and Nutrition Examination Survey (NHANES-III, 1988–1994) [219,220,221,222]. In a Danish study, low levels of vitamin D were strongly correlated with winter SAD symptoms among adolescent girls and elderly women [205]. A high prevalence of SAD along with low vitamin D levels among the elder population from reduced subcutaneous production, intestinal absorption in elderly adults, and limited sun exposure was reported [219]. Vitamin D status was evaluated amongst post-stroke depressed participants in a study that identified a clear variation in depression prevalence during months of decreased sunlight in China (December to May) [223]. In another study that studied the link between SAD and bipolar syndrome among 442 participants, the results revealed a 46% prevalence of vitamin D deficiency. There was a significant difference between the winter and summer months [224,225].

There is a significant correlation between vitamin D and SAD. However, it is unclear whether vitamin D deficiency is the result or the cause [205,226,227,228]. Since there is a direct correlation between vitamin D deficiency and decreased moods/depression symptoms, the association between vitamin D supplementation and SAD shows potential for treatment of symptoms [205,226,227,228] Maintenance of serum 25-hydroxyvitamin D levels during the predominantly affected seasonal increase in depressive symptoms has been explored [208].

#### 4.3.3. Vitamin D Supplementation and SAD

Numerous studies have been conducted to evaluate this association and how vitamin D supplementation can be used as a treatment for MDD [184,227,229,230,231,232]. Results reported from these studies are inconsistent and the evidence that vitamin D can be used as a therapeutic tool for depression is limited. In a study by Schild et al. [231], in a double-blind randomized control trial, female healthcare workers (*n* = 43) experiencing moderate SAD symptoms were randomly assigned a supplementation of 70 IU of vitamin D or an oral placebo over a 12-week winter season trial [231]. Data collected using the SAD and seasonal pattern effective questionnaires identified changes in mood, weight, appetite, and sleep, with no significant improvement in SAD symptoms. In comparison, supplementing vitamin D via intravenous injections have shown significant improvement in the treatment of SAD in a dose-dependent manner [190,226]. In an RCT, participants (*n* = 120) with depressive symptoms were injected with different doses of vitamin D, and significant improvements in depressive symptoms were observed [190]. In this study, the participants were allocated into three groups; one group received an injection of 150,000 IU of vitamin D, the second group received an injection of 300,000 IU of vitamin D, and the last group (control) received a placebo injection [184]. Those who received 300,000 IU of vitamin D exhibited higher levels of improvement in depression symptoms in comparison to the group that was injected with 150,000 IU, while no significant improvement was observed in the patients who had the placebo injection. Authors suggested that the beneficial effects of vitamin D in SAD patients are dose dependent. Moreover, it clearly showed that vitamin D exhibited favorable effects when given in pharmacological doses. It may also explain why previous studies did not show the therapeutic effects of vitamin D when given in physiological doses.

Although there is an inverse association between dietary vitamin D and the prevalence of SAD, the association between supplemental vitamin D and the prevalence of SAD is unclear. A strong link has been observed between depression symptoms, serum vitamin D levels, and unhealthy eating habits [205]. Healthier eating patterns are associated with lower levels of depression symptoms [200,207]. Despite limited investigation to assess the link between dietary and supplemental vitamin D intake and SAD, the results are inconclusive, while most of the evidence indicates vitamin D supplementation has little or no effect [207,231]. Both dietary and supplemental vitamin D have been shown to effectively increase and maintain vitamin D serum levels [208,233].

### 4.4. SAD Prevention and Treatments: Light Therapy

Seasonal affective disorder was recognized by the American Psychiatric Association in 1987 [234]. The theory states that as the days get shorter during autumn- and wintertime, especially those of northern latitudes, this can affect plasma melatonin concentrations that can ultimately result in symptoms that are collectively known as SAD. Light therapy can be utilized as a treatment for therapeutic and physiological purposes, including SAD [209,212,213,232,234,235]. Therapy using concentrated beams of ultraviolet light was discovered in 1903, and Niels Ryberg Finsen received a Nobel Prize for it [236]. Rosenthal, one of the pioneers in the field, found that applying 2500 lux full spectrum light in the morning or evening hours for about five to six hours a day reversed the symptoms of seasonal depression such as fatigue, sadness, overeating, carbohydrate craving, and weight gain in various studies [26,237,238,239,240]. Light therapy was effective after two to four days of exposure. Authors suggested the suppressive effect of the light on melatonin can be the underlying mechanism, but further research was needed [237]. Today, light therapy is considered the first choice when treating SAD [209,234,241,242]. Although many studies were conducted to improve the outcome of light therapy, whether natural light could also be utilized as a therapeutic tool was unclear. Wirz–Justice et al. compared a one (1) hour walk for natural light therapy vs half an hour of light therapy (2800 lux) for one week in 1995 [242]. The outcome showed the artificial light did not help with melatonin or cortisol levels while natural light did exhibit higher melatonin secretions. Authors from this study suggested that natural light is an alternative to artificial light. In another early study, Partonen et al. [218] measured melatonin and serum 25-hydroxyvitamin D3 concentrations before and after exposure to cool white, fluorescent light (3300 lux) in sixteen patients diagnosed with SAD and thirteen non–SAD individuals. They were exposed for fifteen (15) minutes or one (1) hour in the morning. After exposure, the concentrations of melatonin and vitamin D did not change even though there were reports of fewer depressive symptoms and a reduction of sleepiness in the SAD group. No notable differences between the SAD group and the non–SAD group were observed [218]. Light therapy is expected to adjust abnormal melatonin levels initiated by inadequate sunlight which causes a shift in the timing (phase) of circadian rhythms [238,241,243,244]. Theoretically, light therapy in the morning has the most beneficial effect [245] since it prevents a delay in circadian rhythms. The effect of the timing and the dose of light treatment was the focus of several studies; in a clinical study, 96 participants were exposed to a bright light of 6000 lux for one-and-a-half hours a day for four weeks [245]. Participants received three distinctive treatments: morning light time, evening light time, and a morning placebo time. The morning therapy was most beneficial for complete remission compared with the other treatments. In another study, participants were exposed to 10,000-lux light therapy for 30 min for four days at different times of the day: morning, afternoon, and evening [243]. Two additional groups were added to receive light therapy in the morning for two days and then two days in the evening but also interchanged those times. The timing of light therapy did not impact the effects of light therapy [243]. In another study, plasma melatonin concentrations in participants who suffered from SAD were measured. Light therapy (10,000 lux) was applied for 30 min for 10–14 days [246].

The effect of the timing of light therapy (in the morning after awakening vs. two hours before bedtime) was also examined. Morning light therapy created a melatonin rhythm in advance, but evening light therapy only delayed the melatonin rhythm. Although the depression ratings were alike regardless of the timing, the anti-depressant effect of the light was significant when applied in the morning only [246]. The discrepancies in these studies can be explained by the difference in duration, intensity, and the timing utilized in these studies. In one study, using a full spectrum light therapy room, different applications of light therapy were tested [247]. Participants (*n* = 50) diagnosed with seasonal affective disorder received light therapy for an hour and a half to two hours a day, Monday through Friday, for over 10 days. SIGH-SAD was used to gauge the SAD baseline throughout the study. The results showed that individuals had decreased depressive symptoms that lasted for thirty days [247]. This intervention supported the idea that a light therapy room can potentially be applied in the treatment of SAD like portable bright light therapy. More recently, in a randomized controlled trial, a cohort of 62 participants were exposed to either BROAD light therapy 6 h per day in a room inside their home or exposed to a 10,000 lux SAD light box for 30 min per day [244]. Both methods were similarly effective, with improved SAD symptoms among the participants. However, the authors concluded that BROAD light therapy was less confining than standing in front of a light box for 30 min. Wavelength is also a determining factor in light therapy which has been the focus of more recent studies [213,248,249,250]. Glickman et al. [248] examined short wavelengths in blue LED and red LED light therapy with patients diagnosed with SAD. Patients were exposed to light in the morning for 45 min over three weeks and completed the Structured Interview Guide for the Hamilton Depression Rating Scale (SIGH-SAD) survey weekly. The narrow bandwidth blue light at 607 μW/cm^2^ decreased SIGH-SAD scores more than red-light therapy considerably. In contrast, in another study, 45 participants who were suffering from SAD received 30 min of light therapy for five days; 21 participants received broad wavelength white light and 24 participants received narrow-band blue light; the study showed no difference between the lights with two wavelengths [250]. A recent systematic review and meta-analysis of RCTs suggested no indication for the effectiveness of blue-light conditions compared to inactive conditions; low intensity red-light therapy furthermore showed no differentiation with active conditions, as did high intensity white light. The effectiveness of blue-light therapy in the treatment of SAD lacks evidence and more research is needed [213]. In a more recent study, Meesters et al. [250] compared white-light treatment (*n* = 21) with blue-light treatment (*n* = 24) among 45 participants with exposure to 30 min sessions over five consecutive days. There was no statistical significance between the two study groups with changes in mood levels, and both white- and blue-light methods were considered equally effective.

Although the beneficial effect of the light therapy is evident, the underlying mechanisms are still unclear. While the wavelength of the light therapy is still debatable, new propositions for the mechanisms of action, including intrinsically photosensitive retinal ganglion cell (IpRGC) photoreceptors which play a major role in the regulation of sleep/alertness and mood, are now opening windows for further studies. A recent study by Carmel et al. compared different light therapies with specific wavelengths in fat sand rat models to treat SAD [250]. Bright wide spectrum (3000 lux, wavelength 420–780 nm, 5487 K) and blue (1300 lux, wavelength 420–530 nm) and red light (1300 lux, wavelength range 600–780 nm) were applied along with various behavioral tests. Compared to red light, intense wide-spectrum white light and blue light improved depression-like behavior equally. The authors suggested that blue light is just as effective as wide spectrum bright light in the treatment of SAD. Moreover, ipRGCs are intertwined with light therapy and the core of SAD [249].

Individuals with blindness and severe visual impairment (VI) have shown a greater risk of developing SAD [212]. Individuals diagnosed with VI are also more likely to have issues with mood, sleep, and circadian rhythm from a deficiency of sunlight [212]. To investigate the connection between light therapy and photoreceptors, a study examined how light therapy affects individuals with visual impairment (VI) and blindness who experience seasonal affective disorder [212]. Participants with blindness and VI experienced improved mood and sleep after applying six weeks of light therapy for thirty (30) minutes in the morning [212]. In another study, using bright light therapy with SAD individuals through their ear canal via utilizing the photoreceptors was examined [251]. Although it was a small cohort (*n* = 13), 76.9% of the patients obtained full remission on their Hamilton Depression Rating Scale (HAMD-17) [251]. Further research is needed to reveal the mechanisms by which photoreceptors influence the outcome of light therapy and ultimately reduce the SAD symptoms. Monoamine oxidase A (MAO-A), a mitochondrial enzyme, is a key regulator of serotonin, emotion, sleep, and appetite. Recent studies showed elevated MAO-A levels in individuals with non-seasonal major depression [252]. In one study, MAO-A levels in 24 SAD and 27 healthy individuals were examined [211]. The MAO-A levels were analyzed through a positron emission tomography (PET) scan that was completed before treatment in the fall/winter, after treatment, and again in the spring/summer. Bright light therapy (BLT) of 10,000 lux was applied for 30 min in the morning over a three-week span. Similarly, healthy individuals were exposed to light therapy with an intensity of either 10,000 or 400 lux. MAO-A levels in the healthy patients had declined before treatment, and again in spring/summer. There was a reduction in the MAO-A levels in response to the BLT in the SAD patients; however, the MAO-A levels in the SAD patients were not affected by seasonal change. The healthy individuals showed no difference from either light therapy. The authors concluded that the intensity of light therapy is a determining factor in the results of the BLT [211]. Although the mechanisms by which light therapy affects SAD patients are still unclear, the favorable effects of light therapy on depression have been linked to interleukin (IL-6), which is a pro-inflammatory cytokine that has been studied extensively [253,254,255,256,257]. Other pro-inflammatory cytokines include sIL-6R, and 2IL-2R may also play a role in SAD. One study examined whether light therapy has an immunomodulatory effect on SAD patients and examined plasma levels of IL-6, sIL-6, and sIL-2R [210]. The study included 15 SAD patients and 15 healthy individuals. Light therapy was applied with cool white, fluorescent light at 10,000 lux for 30 min early in the morning for two weeks. Plasma levels for immune inflammatory markers were evaluated before and after treatment in the fall/winter season and in March with both groups. The immune inflammatory markers showed an increase in IL-6, IL-6 × sIL-6R, and sIL-2R during the fall/winter season in SAD patients; nonetheless, the outcome of light therapy for two weeks had no effect on plasma levels, while nine of the SAD patients did benefit from light therapy [210]. Further study is needed on other immune inflammatory markers associated with patients with SAD.

The innovation of light therapy has been one of the greatest achievements in the treatment of SAD patients. The mechanisms by which light therapy reduces SAD symptoms received considerable study. Suppression of melatonin due to insufficient exposure to sunlight was the origin of the concept of light therapy as a therapeutic approach [245]. Afterward, other mechanisms including mitochondrial enzymes, photoreceptor pathways, and immune inflammatory markers were proposed [210,211,212,249,251]. Although the favorable effect of light therapy is well documented, the timing, dose, wavelengths, and different applications of light therapy for the treatment of SAD are still open for further investigation [213,238,243,246,247,248,250].

## 5. Summary

Depression is a debilitating mental disorder, ranked within the top three causes of burden of disease worldwide and expected to increase by 2030 [4,8]. Research in the field of nutritional psychiatry has faced significant challenges in comparing studies due to the diverse range of study designs employed by researchers. These variations include differences in intervention regimens, participant demographics, measured parameters, and study designs, making it difficult to draw direct comparisons. B vitamins, particularly B1 (thiamin), B2 (riboflavin), B3 (niacin), B5 (pantothenic acid), B6 (pyridoxine), B9 (folate), and B12 (cobalamin), play crucial roles in neurological function and mood regulation. Deficiencies in these vitamins have been linked to an increased risk and incidence of depression. However, research on the therapeutic effects of dietary B vitamin intake on depression has been limited, with most studies focusing on vitamin supplementation. Studies on individual B vitamins such as thiamin, riboflavin, niacin, and folate have shown promising results in improving depressive symptoms when supplemented. Thiamin deficiency has been associated with mood disorders, and supplementation has shown improvements in depressive symptoms. Riboflavin intake has been inversely associated with depression, while niacin supplementation has improved symptoms of anxiety, irritability, and depression. Folate deficiency has been linked to increased vulnerability to depression, and supplementation has shown antidepressant-like effects.

Vitamin C, an essential antioxidant, is also implicated in mood regulation. Research suggests that vitamin C deficiency may lead to heightened depressive symptoms and that supplementation can alleviate these symptoms. However, the exact mechanisms linking vitamin C deficiency to depression are not fully understood. Seasonal Affective Disorder (SAD), categorized as a MDD with seasonal patterns, manifests through both typical depressive symptoms and atypical vegetative symptoms. Extensive research has explored the link between vitamin D status and SAD, suggesting that insufficient sunlight exposure during autumn and winter may contribute to its onset. Individuals with vitamin D deficiency are reportedly 3.5 times more prone to developing depression. Factors influencing vitamin D levels include skin pigmentation (melanin), sunscreen usage, atmospheric conditions such as cloud cover, seasonal variations, and individual characteristics such as age, obesity, gastrointestinal disorders, dietary habits, supplement intake, and medication interactions. Recent studies investigating the impact of vitamin D supplementation on treating SAD have yielded inconsistent and diverse results due to variations in experimental methodologies. While some research has indicated a positive correlation between light therapy, photoreceptor activity, and SAD, further investigation is warranted to validate these findings across different settings. Current research endeavors focus on exploring various aspects of light therapy, including wavelengths, dosage, and timing, to offer viable alternatives for managing SAD effectively. Additionally, there remains a need for deeper exploration into the underlying mechanisms of SAD, with potential avenues including the role of photoreceptors, mitochondrial enzymes, and immune inflammatory markers, warranting future investigation. In conclusion, nutritional approaches, including dietary and supplemental vitamins, offer promising avenues as preventative and therapeutic tools. B vitamins, particularly thiamin, riboflavin, niacin, and folate, show potential in improving depressive symptoms. Vitamin C deficiency may exacerbate depressive symptoms, while research on vitamin D suggests a link between sunlight exposure, deficiency, and SAD. However, inconsistencies in study methodologies and a limited understanding of underlying mechanisms necessitates further investigation. Future research should focus on elucidating mechanisms of action and refining intervention strategies to effectively utilize these vitamins in mood disorder management, considering individual variability in nutrient status and dietary habits.

## 6. Future Directions

Research on the preventative and therapeutic impacts of dietary B vitamin intake on depression remains relatively restricted, primarily centered on vitamin supplementation. However, there is a critical need for more comprehensive investigations into the influence of dietary vitamin B intake on depression at a mechanistic level and in a dose-dependent manner. Understanding the complex mechanisms through which these vitamins interact with neural processes could provide valuable insights into their efficacy in managing depressive symptoms. Furthermore, exploring the role of nutrigenetics and nutrigenomics in modulating the effects of vitamins on depression represents a promising field that warrants significant attention. Unraveling how individual genetic variations influence an individual’s response to dietary interventions with B vitamins could help to personalize nutritional strategies for combating depression. This interdisciplinary approach holds promise for elucidating the complex interplay between genetics, nutrition, and mental health outcomes. Overall, while there is evidence supporting the role of B vitamins and vitamins C and D in the prevention and treatment of depression, further research is needed to elucidate their mechanisms of action and to determine optimal intervention strategies. Additionally, considering individual variations in baseline nutrient status and dietary habits is crucial when evaluating the therapeutic potential of these vitamins in mood disorders.

## Figures and Tables

**Table 1:** A comparison between seasonal Affective Disorder and Depression.

Seasonal Affective Disorder	Vs.	Depression
Reduced sunlight, circadian rhythm, latitude, vit D deficiency, melatonin overproduction, decreased serotonin levels, lower dopamine production	Etiology	Genetic factors; dietary factors; deficiency of neurotransmitters noradrenalin, serotonin, and dopamine; hypothyroidism; Cushing’s syndrome.
Similar	Symptoms	Similar
Possible weight gain	Weight gain	Possible weight gain/loss
More likely in the fall or winter	Timing	No specific time
Responding	Antidepressants	Responding
Responding	Light Therapy	Unclear. Needs more study.
